# Metastasis of Mumps

**Published:** 1881-03

**Authors:** C. W. Robbins


### NOTES FROM PRIVATE PRACTICE.

### ARTICLE VII.

*Metastasis of Mumps.*

This is a rare complication of that disease, and I am for that reason induced to send the report of a case occurring in my practice.

Mrs. B., age twenty-three, had had mumps for one week. The swelling was extreme. The brunt of the attack was borne by the sub-maxillary glands. The parotid and sub-lingual glands were moderately enlarged. She was then seized with a chill, followed by vomiting and an intense headache. This was at 4 p. m. When seen by me at 10 p. m. the cephalalgia was so severe that she could not lie still in her bed; pulse 112, sharp and quick, temperature 101, tongue coated with fur. The swelling in the salivary glands had greatly diminished since the onset of inflammation of brain, and next morning had entirely disappeared. Patient then presented the following symptoms: Intolerance of light and sound, delirium, pain over the whole of vertex, reversed action of pupils, restlessness and insomnia; pulse, 114 sharp and quick; temperature, 101.4; tongue, coated; bowels, obstinately constipated. This state of things continued during the day up to 10 o'clock. When I saw the patient next morning she was then out of danger. The pain had gone and she was perfectly rational, in fact, well; the disease leaving her suddenly in the night. That afternoon she again had a rise of temperature and rapid pulse, 100, and I looked for a return of meningeal trouble, but it proved to be a return of the parotitis. The glands on one side were moderately enlarged for two days, and then permanently subsided. The treatment adopted from the first was chloral hydrate, gr. 20, brom. potassium, gr. 40, every three hours, with ice to the head constantly, and several doses of Rochelle salts, none of which acted, however. I should have given her a dose of croton oil last morning had she not been almost entirely free from any symptoms of meningitis. The reversed action of pupils, as I term it, consists of the pupil contracting when shaded from the light with the hand, and dilating immediately on exposure to light by withdrawing the hand. This is a symptom which I noticed once before in a case of meningitis (*basilar traumatica.*) I have never seen the symptoms noticed in the books, and have not met anyone who has ever noticed it.

C. W. ROBBINS, M. D.

---

A SANITARY CONVENTION will be held at Battle Creek, Michigan, March 29 and 30, 1881, under the auspices of the State Board of Health. In accordance with invitation received from citizens of that place, arrangements having been made by a local committee, acting with a committee of the State Board of Health.

There will be sessions the first day at 3 p. m. and 7:30 p. m.; on the second day at 9:30 a. m., 2:30 p. m., and 7:30 p. m.

During each session of the convention there will be one or more addresses on papers on some subject of general interest pertaining to public health, each paper to be followed by a discussion of the subject treated.

The officers chosen by the committee are as follows: Rev. D. F. Barnes, President; Hon. George Willard, Prof. Z. C. Spencer, Mr. L. McCoy, Rev. Dr. Sidney Corbett, Edward Cox, M.D., Judge B. F. Graves, Vice Presidents; J. H. Kellogg, M.D., Secretary.

Manufacturers of and dealers in all kinds of sanitary apparatus or appliances are invited to send specimens of their articles for exhibition at these conventions.

